# A randomized, controlled trial to investigate cognitive behavioral therapy in prevention and treatment of acute oral mucositis in patients with locoregional advanced nasopharyngeal carcinoma undergoing chemoradiotherapy

**DOI:** 10.3389/fonc.2023.1143401

**Published:** 2023-06-07

**Authors:** Li-li He, Shuai Xiao, Cui-hong Jiang, Xiang-wei Wu, Wen Liu, Chang-gen Fan, Xu Ye, Qi Zhao, Wen-qiong Wu, Yan-xian Li, Hui Wang, Feng Liu

**Affiliations:** ^1^ Department of Radiation Oncology, Hunan Cancer Hospital and The Affiliated Cancer Hospital of Xiangya School of Medicine, Central South University, Changsha, Hunan, China; ^2^ Hunan Key Laboratory of Translational Radiation Oncology, Hunan Cancer Hospital and The Affiliated Cancer Hospital of Xiangya School of Medicine, Central South University, Changsha, Hunan, China

**Keywords:** cognitive behavioral therapy (CBT), chemoradiotherapy, nasopharyngeal carcinoma, acute oral mucositis, toxicities

## Abstract

**Purpose:**

Oral mucositis is a common side effect of concurrent chemoradiotherapy (CCRT). This study aimed to determine whether cognitive behavioral therapy (CBT) could help prevent oral mucositis during chemoradiation therapy for locoregional advanced nasopharyngeal carcinoma (LA-NPC).

**Methods and materials:**

Between July 15, 2020, and January 31, 2022, a randomized controlled phase II trial was conducted. Eligible patients (N=282, 18-70 years old) with pathologically diagnosed LA-NPC were randomly assigned to receive CBT or treatment as usual (TAU) during CCRT (computer-block randomization, 1:1). The primary endpoints were the incidence and latency of oral mucositis.

**Results:**

The incidence of oral mucositis was significantly lower in the CBT group (84.8%; 95% confidence interval [CI], 78.7%-90.9%) than in the TAU group (98.6%; 95% CI, 96.6%-100%; P<0.001). The median latency period was 26 days and 15 days in the CBT and TAU groups, respectively (hazard ratio, 0.16; 95% CI, 0.12-0.22; P<0.001). CBT significantly reduced ≥ grade 3 oral mucositis (71.9% vs. 22.5%, P<0.001), dry mouth (10.8% vs. 3.7%, P=0.021), dysphagia (18% vs. 5.1%, P=0.001), and oral pain (10% vs. 3.6%, P=0.034) compared with TAU. Patients receiving CBT and TAU during CCRT had similar short-term response rates.

**Conclusions:**

CBT reduced the occurrence, latency, and severity of oral mucositis in patients with LA-NPC during CCRT.

## 1 Introduction

Nasopharyngeal carcinoma (NPC) is a malignant tumor with the highest incidence in southern China (1, 2). Concurrent chemoradiotherapy (CCRT) is a major component of curative therapy for locoregional advanced nasopharyngeal carcinoma (LA-NPC) (3, 4); however, it can lead to toxic side effects. The most common complication of NPC related to chemoradiotherapy (CRT) is oral mucositis (OM) (5), and its incidence in patients with NPC ranges from 85% to 100% (6–8). OM commonly reduces the nutritional status and quality of life of patients owing to pain and dysphagia in the mouth (9). Patients with severe oral mucositis (SOM; classified into 3-4 grades according to World Health Organization [WHO]) frequently experience an interruption of treatment and prolonged treatment, both of which negatively affect treatment outcomes (10–13).

Basic oral care is a good clinical practice for oncology patients with mucositis (14). Unfortunately, there is no standard treatment for the prevention of OM. Topical agents (15), oral drugs (16–19), and intravenous drugs (20) have all been studied for the prevention and treatment of OM caused by chemotherapy/radiotherapy; however, none of them can be used as a standard treatment. Photobiomodulation is effective in preventing OM caused by CCRT; however, it can cause cancer in the long run (21, 22).

Consequently, there is an urgent need to develop a feasible and effective management method to take precautions against OM in LA-NPC patients undergoing CCRT. Cognitive behavioral therapy (CBT) has already been confirmed to be an effective psychological treatment to avoid or decrease the occurrence of adverse effects in patients with proven malignancies (23–26). In our previous study, we found that the combination of CBT and CRT significantly reduced acute OM in patients with LA-NPC (27).

To the best of our knowledge, no randomized clinical trials have investigated CBT to prevent and relieve acute chemoradiotherapy-induced OM and improve the survival quality of LA-NPC. Therefore, we performed a randomized controlled trial to assess the preventive and therapeutic effects of CBT on OM in patients with LA-NPC treated with chemoradiotherapy.

## 2 Methods and materials

### 2.1 Clinical trial design

This study was a phase II, prospective, randomized, single-center clinical trial. The assay was registered on the chictr.org.cn website (ChiCTR2000034701). This prospective study was approved by the institutional ethics committee and conducted in accordance with the ethical standards formulated in the Declaration of Helsinki. Patients with advanced cancer diagnosed by pathology were selected according to the principles of phase II clinical trial and signed informed consent forms. After checking the eligibility criteria, a computer-generated code was used for randomization. Patients were randomized in a 1:1 ratio to receive either CBT (group A) or treatment as usual (TAU) (group B).

### 2.2 Eligibility criteria

All the cases were diagnosed as NPC by pathology. Patients with the following baseline characteristics were eligible for the study: WHO pathological types II-III, clinical stage III-IVa (8_th_ American Joint Committee on Cancer); 18-70 years old; Karnofsky physical status ≥ 70; absence of significant oral disease; undergoing 1 to 3 cycles of induction chemotherapy (IC); and normal routine blood cell tests (the total number of leukocytes ≥4.0 × 10^9^/L, absolute neutrophil count ≥1.5 × 10^9^/L, hemoglobin ≥90 g/L, and platelets ≥100 × 10^9^/L), hepatic, and renal function tests. Patients were excluded if they had a history of prior radiation therapy (RT), secondary primary malignant tumor, evidence of distant metastasis, OM or recurrent OM prior to CRT, gingivitis or stomatitis, severe life-threatening illness, psychological or mental health conditions (such as suicidal tendency), and pregnancy or lactating.

### 2.3 Baseline assessment

Detailed case history of patients with NPC was recorded prior to their treatment. Routine physical examination, hematology and biochemical indices, Epstein-Barr virus (EBV) DNA copies, electro-nasopharyngoscope, nasopharynx and neck enhanced magnetic resonance imaging (MRI), chest enhanced computed tomography (CT), abdominal ultrasound, whole body bone SPECT imaging, and dental and nutritional status were also assessed in our study before IC.

### 2.4 Radiotherapy

Mask immobilization was performed in all patients using CT simulations and CT-based planning. The CT simulation was conducted one week after the IC cycle. All patients underwent enhanced MRI before and after IC.

All patients were treated with intensity-modulated radiation therapy (IMRT) at a dose of 70.4 Gy/32 fx and 72.6 Gy/33 fx to the gross tumor volume of the nasopharynx (GTVnx) in stage T1-2 and T3-4 disease, respectively. A total dose of 69.96 to 72.6 Gy was administered to the gross tumor volume of the lymph nodes (GTVnd). Doses prescribed for high-risk subclinical lesions (planning target volume 1, PTV1) and lower-risk subclinical disease (planning target volume 2, PTV2) were 60.06 to 64 Gy/32 to 33 fx and 50.96 to 56.0 Gy/26 to 28 fx, respectively. RT was administered daily from Monday through Friday for 32–33 days. It is important to note that the normal tissue doses were designed according to Radiation Therapy Oncology Group (RTOG) 0615 (28) and RTOG 0225 protocols (29). In our study, onboard image guidance was performed prior to the first five treatments and then weekly thereafter.

### 2.5 Chemotherapy

The IC regimen consisted of TPF (docetaxel at 60 mg/m^2^, d1, intravenous infusion, plus cisplatin at 60 mg/m^2^, d1, intravenous infusion, and 5-fluorouracil at 600 mg/m^2^, d1-d5, intravenous 120-hour infusion) or TP (docetaxel at 75 mg/m^2^, d1, intravenous infusion, plus cisplatin at 75 mg/m^2^, d1, intravenous infusion) administered every 3 weeks. Patients were prescribed CCRT with cisplatin alone (80–100 mg/m^2^, d1, intravenous infusion) every 3 weeks. All patients planned to receive CCRT, except those who declined treatment or experienced severe adverse events. According to previous studies (30, 31), the cut-off value of cumulated concurrent cisplatin dose was 200 mg/m^2^.

### 2.6 Cognitive behavioral therapy

The large body of empirical data from the work of Ellis (1962) and Beck (1976) (32) and the manual written by Beck et al. (33) supports the efficacy of CBT in treating psychological conditions and associated adverse events. A special treatment plan was designed based on previous studies (24, 34, 35) and the psychological characteristics of patients with NPC. Behavioral, cognitive, and educational strategies were combined into the intervention. The participants in the CBT group received six sessions along with concurrent chemoradiotherapy. The CBT method used in this study is the same as that used in our previous study (27).

The intervention was led by an oncologist doctor, and a multidisciplinary team including a psychotherapist, two oncologists, and two nurses had a 3-day training course. In a group of six participants, CBT was administered once a week in 45 minutes sessions for 6 weeks during concurrent chemoradiotherapy. Prior to the study, all evaluators and therapists received rigorous and uniform training, following Standard Operating Procedures (SOPs) to ensure the quality of this study. To ensure protocol adherence, sessions and scripts were recorded. We randomly selected and assessed the fidelity of the recordings and provided feedback to the psychotherapist. Weekly themes and the main content of the CBT sessions in the present study were the same as those in our previous article (27).

### 2.7 Treatment as usual

All patients were treated with TAU according to the standards of the oncology radiotherapy department. TAU consists of irregular intervals of educational sessions that include information on health, nutrition, and psychology and provides explanations tailored to the patient or family’s problems.

### 2.8 Concomitant medication

All patients were given conventional oral health guidance and education. All patients underwent oral cleaning immediately after eating. From the first day of chemoradiotherapy, oral cleaning immediately after eating was administered until the entire radiotherapy course was completed. Other medicines for oral mucositis including hormones and antibiotics were not prescribed for patients with grade 1-2 oral mucositis. Sodas and antifungal agents were used in patients with oral cavity fungal infections. Patients could withdraw from the trial if they had grade 3 or higher oral mucositis or if they did not wish to continue the study.

### 2.9 Evaluation

According to the National Cancer Institute (NCI) Common Terminology Criteria for Adverse Events (CTCAE) version 5.0, oral mucositis is defined as a disorder characterized by ulceration or inflammation of the oral mucosa. The grading criteria of oral mucositis as per CTCAE5.0 were as follows: 1 = Asymptomatic or mild symptoms, intervention not indicated; 2 = Moderate pain or ulcer that does not interfere with oral intake, modified diet indicated; 3 = Severe pain interfering with oral intake; 4 = Life-threatening consequences, urgent intervention indicated; 5 = Death. Oral pain is defined as a sensation of marked discomfort in the mouth, tongue, or lips. The grading criteria of oral pain as per CTCAE5.0 were as follows: 1 = mild pain; 2 = moderate pain, limiting instrumental activities of daily living (ADL); and 3 = severe pain, limiting self-care ADL. Acute oral mucosal toxicity and pain during treatment were carefully observed and assessed daily. The time to the development of grade 3 mucositis was recorded on day 1 of radiotherapy.

Both groups were evaluated for anxiety and depression by a trained psychotherapist using the Hospital Anxiety and Depression Scale (HADS) at baseline (T1) and the end of CCRT (T2). The HADS is a 14-item self-administered questionnaire that measures the symptoms of anxiety (HADS-A) and depression (HADS-D) in patients with somatic illness. The possible scores ranged from 0 to 21 for anxiety and 0 to 21 for depression. This scale has been widely used in cancer research (27, 36). A higher score indicates a more severe level of anxiety and/or depression.

Three months after the completion of chemoradiotherapy, the patients underwent physical examination, nasopharyngoscopy, and MRI to assess tumor response, which was classified according to the Response Evaluation Criteria in Solid Tumors (version 1.1) (37).

### 2.10 Outcomes

The incidence of oral mucositis and latency to oral mucositis during this study were used as the primary efficacy endpoints. The severity of oral mucositis and accompanying symptoms (e.g., dry mouth, dysphagia, and oral pain) were used as secondary efficacy endpoints.

### 2.11 Sample size

PASS v11 software was used to calculate the sample size. Two-sided alpha was used in this study. Based on previous randomized studies assessing oral mucositis during chemoradiotherapy for locally advanced NPC, the incidence of ≥ grade 3 oral mucositis ranged from 61.6% to 74.0% (17, 38). The study was designed to detect a 25% difference in the incidence of grade 3 mucositis, assuming an incidence of 70% in the control group. A minimum sample size of 224 (112 in each group) was required for a power of 80% and a significance level (alpha) of 0.05. To allow for a 10% loss rate, the total sample size required in each group was at least 246. One-way ANOVA and chi-square tests were used to compare measurement data and count data between the two groups, respectively.

### 2.12 Statistical analysis

Fisher’s exact test was used to compare the incidence of different degrees of oral mucositis, and 95% confidence intervals (CIs) were calculated. The median time of occurrence of oral mucositis (latency) and 95% confidence interval (CI) was estimated using the Kaplan-Meier method. To compare latencies, the Cox proportional hazards model was used to calculate the hazard ratio and 95% CI values. Secondary efficacy endpoints and toxicities were compared using a 1-way analysis of variance and Fisher’s exact test. For HADS scores, the total and subscale scores of each measure at the two time points (T1 and T2) were analyzed using an independent samples t-test. All statistical tests were two-sided, and statistical significance was set at P < 0.05. All statistical analyses were performed using the commercial software package SPSS version 25.0 (IBM Corporation, Armonk, NY, USA).

## 3 Results

**Figure 1:**
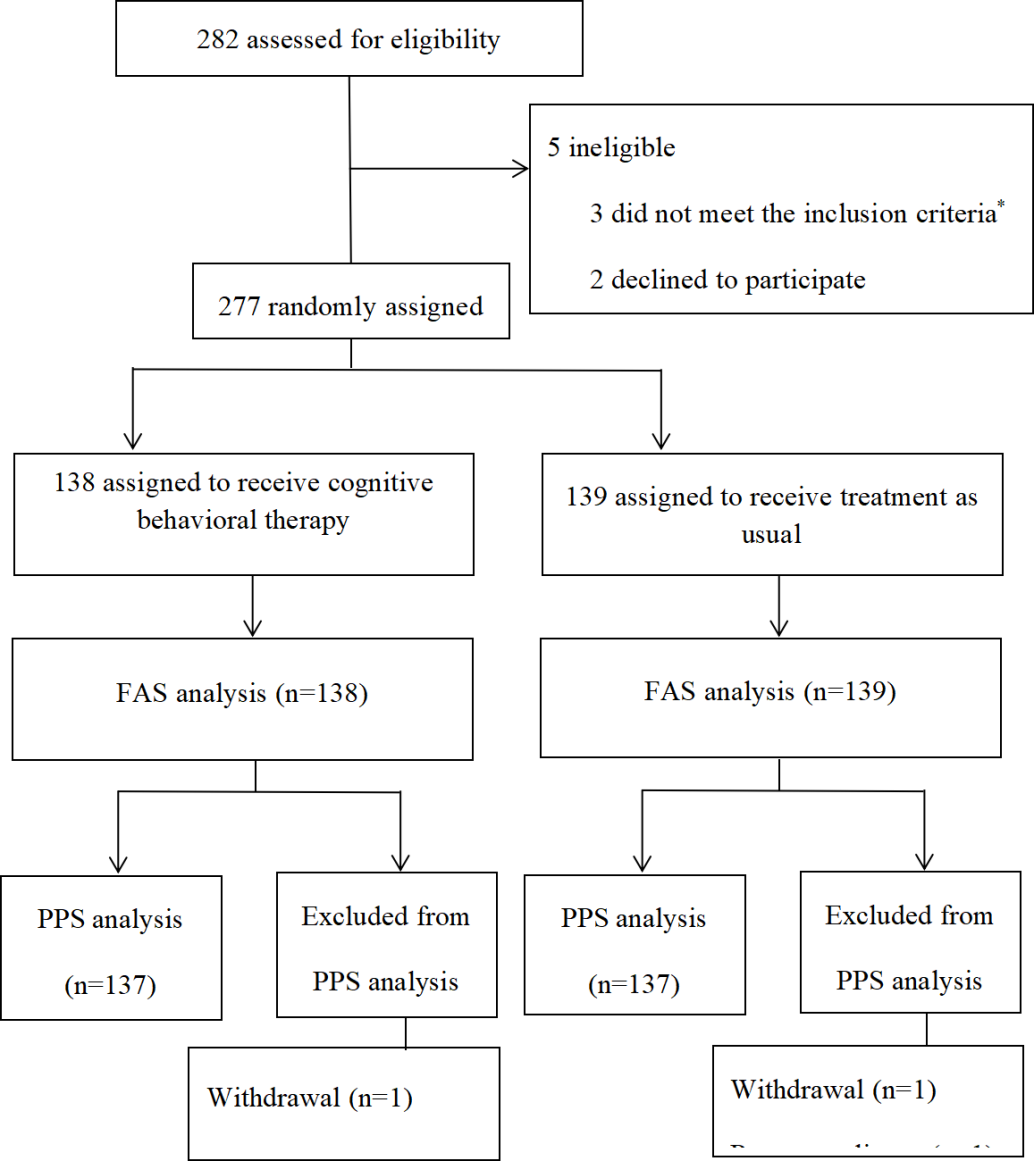
Patients included in the study. *Three patients with distant metastasis, two patients without adequate hematological function, one without adequate renal function, two with hepatotoxicity, and one patient with heart disease. FAS = full-analysis set; PPS = per-protocol set.

From July 15, 2020, to January 31, 2022, 282 patients with LA-NPC were randomly assigned to one of the two study groups (**Figure 1**). A total of 138 patients in the CBT group received cognitive behavioral therapy, and 139 patients in the TAU group received treatment as usual. The clinical characteristics of the two groups were relatively balanced. **Table 1** provides additional information.

**Table 1:** Baseline characteristics.

Characteristics	CBT group	TAU group	
	No. of patients (%)	No. of patients (%)	P value
Total	138	139	
Age, y			
Median	51	52	
Range	18-70	23-70	
Sex			0.358
Male	105 (76.1)	99 (71.2)	
Female	33 (23.9)	40 (28.8)	
Karnofsky scale			0.414
90-100	122 (88.4)	127 (91.4)	
70-80	16 (11.6)	12 (8.6)	
Smoking status			0.572
Never-smoker	40 (29.0)	38 (27.3)	
Cigarette smoker, pack-years			
< 10	22 (15.9)	29 (20.9)	
≥ 10	76 (55.1)	72 (51.8)	
EBV DNA			0.966
≥400 copies/mL	91 (65.9)	93 (66.9)	
<400 copies/mL	47 (34.1)	46 (33.1)	
Pathology			0.644
WHO type II	33 (23.9)	30 (21.6)	
WHO type III	105 (76.1)	109 (78.4)	
T category			0.207
T1	5 (3.6)	6 (4.3)	
T2	43 (31.2)	56 (40.3)	
T3	68 (49.3)	51 (36.7)	
T4	22 (15.9)	26 (18.7)	
N category			0.149
N0	6 (4.3)	5 (3.6)	
N1	12 (8.7)	21 (15.1)	
N2	93 (67.4)	77 (55.4)	
N3	27 (19.6)	36 (25.9)	
Disease stage			0.190
III	90 (65.2)	80 (57.6)	
IVA	48 (34.8)	59 (42.4)	
IC regimen			0.654
TPF	129 (93.5)	128(92.1)	
TP	9 (6.5)	11 (7.9)	
Number of cycles of IC			0.690
1 cycle	19 (13.8)	15 (10.8)	
2 cycles	65 (47.1)	71 (51.1)	
3 cycles	54 (41.1)	53 (38.1)	

^*^Three patients with distant metastasis, two patients without adequate hematological function, one without adequate renal function, two with hepatotoxicity, and one patient with heart disease. Abbreviations: FAS = full-analysis set; PPS = per-protocol set

### 3.1 Treatment compliance

One hundred and thirty-seven patients (99.3%) in the CBT group and 137 patients (98.6%) in the TAU group completed at least two cycles of CCRT (**Table 2**). CCRT was suspended owing to severe hematological toxicity, severe vomiting, and patient refusal. One patient in the CBT group withdrew from the trial in the seventh week of CCRT because of severe leukopenia and fatigue. Two patients in the TAU group who could not tolerate the symptoms of severe OM ended the trial early in the sixth week of CCRT. For patients who withdrew from the trial owing to grade 3 or higher oral mucositis, the standard treatment for severe oral mucositis was applied. All patients in the CBT group completed the planned six sessions of CBT. All but one of the participants received the full planned dose of radiotherapy without any treatment delays > 5 days. The patient in the TAU group received 70.4 Gy (97.0%) of the prescribed RT dose (72.6 Gy planned) and treatment was suspended due to severe hematological toxicity. No significant differences were observed in cycles of concurrent cisplatin, cumulative concurrent cisplatin dose, radiation treatment delay, and RT dose completion between the two groups (P=0.525, P=0.403, P=0.684, and P=1.000, respectively).

**Table 2:** Treatment compliance.

Treatment	No.(%)		
	CBT group (n=138)	TAU group (n=139)	All Patients (N=277)
CCRT			
1 cycle of cisplatin	1 (0.7)	2 (1.4)	3 (1.1)
2 cycles of cisplatin	19 (13.8)	25 (18.0)	15 (15.9)
3 cycles of cisplatin	118 (85.5)	112 (80.6)	230 (83.1)
Cumulated concurrent cisplatin dose			
≥ 200 mg/m^2^	128 (92.8)	125 (90)	253(91.3)
< 200 mg/m^2^	10 (7.2)	14 (10)	24 (8.7)
Radiotherapy			
Treatment delay, days			
No	136 (98.6)	135 (97.1)	271 (97.8)
≤ 5	2 (1.5)	4 (2.9)	6 (2.2)
> 5	0 (0)	0 (0)	0 (0)
RT dose completed	138 (100)	138 (99.3)	276 (99.6)

### 3.2 Incidence and severity of mucositis

The incidence of oral mucositis during the study period was 84.8% (95% CI, 78.7-90.9%) and 98.6% (95% CI, 96.6-100%) in the CBT and TAU groups, respectively. CBT significantly reduced the incidence of oral mucositis (P<0.001). The CBT group also had a lower incidence of grade ≥ 3 mucositis than the TAU group (22.5% vs. 71.9%, P<0.001).

According to the protocol, patients could withdraw from the trial if grade 3 or higher oral mucositis developed and if they are unwilling to continue taking the study drug. The most severe grade of oral mucositis involved in this study was Grade 3. Comparing the TAU group with the CBT group, the incidence of grade 1, 2, and 3 oral mucositis was 98.6% versus 84.8% (P<0.0001), 87.1% versus 58% (P<0.0001), and 71.9% versus 22.5% (P<0.0001), respectively (**Table 3**).

**Table 3:** Incidence of different degrees of oral mucositis during the study .

	CBT group		TAU group		
	N	95%CI	N	95%CI	P value
All patients	n=138		n=139		
Grade 1	117 (84.8%)	78.7-90.9	137 (98.6)	96.6-100	<0.0001
Grade 2	80 (58%)	49.6-66.3	121 (87.1)	81.4-92.7	<0.0001
Grade 3	31 (22.5)	15.4-29.5	100 (71.9)	64.4-79.5	<0.0001

CI, confidence interval.

### 3.3 Onset time of mucositis

**Figure 2:**
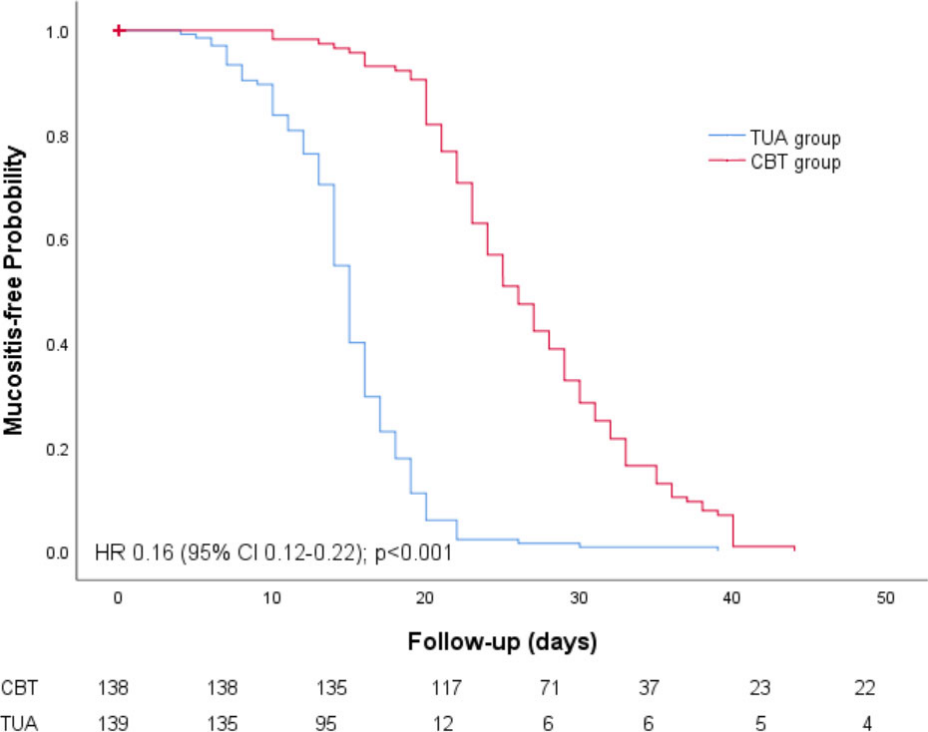
Mucositis-free probability CI, confidence interval; HR, hazard ratio.

The median latency to onset of oral mucositis was 14.7 days (interquartile range, 12.5-17 days) in the TAU group and 22.6 days (interquartile range, 20-30 days) in the CBT group. Such results showed that CBT significantly delayed the occurrence of oral mucositis (P<.0001, **Figure 2**). The mean time to onset of grade 2 mucositis was 25± 5.5 days (range, 15–40 days) and 15.5 ± 4 days (range, 7–22 days) in the CBT and TAU groups, respectively (P = 0.001). The onset time of grade 3 mucositis was also significantly longer in the CBT group than in the TAU group (24.5 ± 6 days (10-36 days) vs. 15 ± 4 days (4-30 days), P = 0.001).

### 3.4 The severity of oral pain

Patients in both groups experienced varying degrees of OM during the treatment (**Table 3**). The CBT group had a lower incidence of grade 3 pain than the TAU group (3.6% vs. 10%, P = 0.034). Thirty-three patients (14 and 19 in the CBT and TAU groups, respectively) were administered topical anesthesia for grade 2 or 3 oral pain.

The incidence of ≥ grade 3 symptoms (dry mouth, dysphagia, and mouth pain) of oral mucositis was recorded in both groups. Comparisons of ≥ grade 3 dry mouth (10.8% vs. 3.7%, P=0.021), dysphagia (18% vs. 5.1%, P=0.001), and oral pain (10% vs. 3.6%, P=0.034) showed that the incidence of OM was more likely to be reduced in the CBT group than in the TAU group. The response rates for the TAU and CBT groups were 98.6% (136/138) and 98.6% (137/139, P=1.000), respectively, and the disease control rates of the TAU and CBT groups were 99.0% (137/138) and 99% (138/139), respectively (P=1.000). These results showed that CBT had no effect on the short-term response rate to chemoradiotherapy, and the effect on long-term efficacy is currently being followed up.

### 3.5 Adverse events

Adverse events were recorded in 136 of the 138 patients (98.6%) in the TAU group and 137 of the 139 patients (98.6%) in the CBT group. Except for oral mucositis, the most common adverse events (≥8%) were leukopenia (8% vs. 8.7%, P=0.814) and neutropenia (8% vs. 6.5%, P=0.654). The incidence of insomnia, fatigue, weight loss, anemia, dry mouth, and serum albumin was significantly lower in the CBT group than in the TAU group. The incidence of other side effects, which were mainly caused by the cytotoxic effects of chemotherapy or radiation therapy, was not different between the two groups (**Table 4**).

**Table 4:** Grade 3–4 acute adverse events.

Grade 3–4 Acute adverse events	CBT group (N=138)	TAU group (N=139)	P-value
Anemia	0 (0%)	6 (4.3%)	0.040
Leukopenia	12 (8.7%)	11 (8.0%)	0.814
Neutropenia	9 (6.5)	11 (8.0%)	0.654
Thrombocytopenia	0 (0%)	4 (2.8%)	0.133
Liver dysfunction	0 (0%)	0 (0%)	–
Nephrotoxicity	0 (0%)	0 (0%)	–
Nausea	0 (0%)	1 (0.7%)	1.000
Vomiting	0 (0%)	2 (1.4%)	0.481
Fatigue	7 (5.1%)	19 (13.7%)	0.014
Dry mouth	5 (3.7%)	15 (10.8%)	0.021
Mucositis	31 (22.5%)	100 (71.9%)	0.001
Dermatitis	0 (0%)	1 (0.7%)	1.000
Dysphagia or odynophagia	7 (5.1%)	25 (18%)	0.001
Oral pain	5 (3.6%)	14 (10%)	0.034
Ototoxicity	0 (0%)	0 (0%)	–
Insomnia	5 (3.6%)	18 (13%)	0.006
Weight loss	5 (3.6%)	16 (11.5%)	0.012
Serum albumin	0 (0%)	6 (4.3%)	0.040

### 3.6 Anxiety and depression

As shown in **Table 5**, there were no significant differences in the mean HADS scores between the CBT and TAU groups at baseline. The HADS scores decreased in both groups at the end of CCRT. Patients in the CBT group showed significantly lower mean total HADS scores and mean HADS scores for depression and anxiety than those in the TAU group at the end of CCRT (P<0.001, P<0.001, and P<0.001, respectively).

**Table 5:** HADS scores among the two groups.

Time	CBT group	TAU group	
	Mean (SD)	Mean (SD)	P value
Baseline (T1)			
Total	18.03 (3.528)	17.53 (3.397)	0.229
Anxiety	9.16 (1.866)	8.96 (2.028)	0.382
Depression	8.85 (1.843)	8.57 (1.622)	0.178
End of CCRT (T2)			
Total	11.19 (1.971)	13.15 (2.680)	<0.001
Anxiety	4.00 (1.120)	5.76 (1.315)	<0.001
Depression	4.09 (1.162)	5.79 (1.443)	<0.001

## 4 Discussion

Acute OM, characterized by oral pain, ulceration, necrosis, and pseudomembrane formation, is a common adverse event associated with radiotherapy. It can also be caused by chemotherapy, and usually occurs 7-14 days after the initiation of drug therapy. The severity of oral mucositis is significantly aggravated when radiotherapy and chemotherapy are concomitantly combined (39–43). Recently, induction chemotherapy followed by concurrent chemoradiotherapy has been widely used to treat LA-NPC (44). Since severe mucositis affects treatment compliance and quality of life, management of oral mucositis induced by chemoradiotherapy is important.

Previous studies have shown the efficacy of CBT in reducing treatment-related adverse events (sleep disorders, fatigue, anemia, weight loss, anxiety, depressive symptoms, etc) in survivors of various cancers, including breast, head and neck, and colorectal cancers (26, 45, 46). Garland et al. showed that CBT significantly improved sleep continuity in patients with cancer (47). In a study by Gielissen et al., fatigue in cancer patients was significantly reduced by CBT, and a positive effect was still observed 2 years after the completion of CBT (48). Treatment outcomes for NPC were influenced by pretreatment and mid-treatment hemoglobin (Hb) levels (49). In the present study, the CBT group showed a lower incidence of anemia than the TAU group, which might have had a positive effect on treatment outcomes.

CCRT following IC improved the prognosis of LA-NPC; however, the incidence of OM remained high in patients with NPC undergoing CCRT. Lv et al. (7) found that the incidence of OM in LA-NPC patients treated with CCRT was 97–98%. Another multicenter randomized trial found that the incidence of OM in patients with NPC undergoing CCRT was 97.1% and the incidence of grade 3-4 OM was 32.1% (50). In our present study, patients in the CBT group had lower incidences of OM (84.8%) and SOM (22.5%), compared to that in the TAU group (98.6% and 71.9%, respectively). These results indicate that CBT can reduce the incidence and severity of OM. The median latency period of OM in the CBT group (22.6 days) was significantly longer than that in the TAU group (14.7 days), suggesting that the onset of OM was significantly delayed by CBT.

CBT can improve malnutrition and consequently reduce the incidence of oral mucositis. ROM had strong association with nutritional status which was strongly related to body weight, serum albumin and hemoglobin levels (51–54). Li et al. (51) reported that body weight loss (BWL) was associated with severe acute oral mucositis in LA-NPC patients treated with CCRT. For patients with BWL ≥ 5%, the risk of ≥ grade 3 OM increased by approximately 4 times. Su et al.’s study showed that (52) severe nutritional impairment was an independent risk factor for grade ≥2 oral mucositis of patients with NPC. A prospective study by Shu et al. (54) demonstrated that malnutrition occurred early and worsened continuously during radiotherapy in patients with NPC. Radiation-induced oral mucositis (ROM) was strongly associated with nutritional status, body weight, and serum albumin levels. Huang et al. (51) reported that systematic nutrition management could significantly reduce grade 3-4 oral mucositis during radiotherapy in patients with LA-NPC. Liang et al. (16) reported that thalidomide (THD) treatment reduced the incidence of OM and degree of weight loss and significantly decreased the incidence of vomiting, nausea, and insomnia. Our retrospective previous study (27) showed that CBT significantly reduced the incidences of grade 3 to 4 acute oral mucositis, as well as anemia and weight loss for patients with LA-NPC underwent CRT. In the present study, the CBT group had a lower incidence of BWL, which may be related to its lower incidence of oral mucositis.

The results of the present study also show that the incidence of ≥ grade 3 insomnia and fatigue in NPC patients was significantly reduced by CBT, which is consistent with the findings of Kangas et al. (24) and Gielissen et al. (48). CBT is effective in reducing anxiety and depression in cancer patients (23, 25, 27). Our results also showed that the addition of CBT to chemoradiotherapy significantly reduced depressive and anxiety symptoms.

To the best of our knowledge, no prospective trial has evaluated the effect of CBT plus chemoradiotherapy on response rates in patients with NPC. The CR rate after chemoradiotherapy for LA-NPC is between 82.8% and 98% (55–57). Our previous retrospective study showed that the CBT group had a significantly higher CR rate than the TAU group. The present study showed that CBT tended to increase the CR rate; however, no statistical difference was observed. Liang et al. indicated that THD resulted in a reduction of the incidence of OM, and had no effect on the short-term efficacy of CCRT in NPC patients (16). Weng et al. reported that antibiotics were effective for treating grade 3/4 radiation-induced mucositis but may have potential adverse effects on the prognosis of NPC patients. Compared with antibiotics, CBT can prevent oral mucositis without reducing the treatment response. Further clinical trials are needed to assess the effects of CBT on the treatment response in patients with SOM.

In the present study, patient compliance with CBT was good. None of the patients withdrew from the trial because they were unable or unwilling to undergo CBT. CBT may improve compliance with physicians’ instructions (including rinsing the mouth, usage of related medication, and nutritional instruction). We did not assess the effect of CBT on compliance with physicians’ instructions in the present study; however, this should be evaluated in future prospective clinical trials. An advantage of CBT is that, as psychotherapy, it does not require oral or intravenous medications. In the present study, no CBT-related adverse events were observed in the CBT group. Other oral drugs (16–19) and intravenous drugs (20) used for prevention and treatment of OM could caused severe adverse events. In a randomized controlled trial by Zheng et al. (17), Shuanghua Baihe tablets (a traditional Chinese medicine) were orally administered to patients with LA-NPC for up to seven weeks during chemoradiotherapy. Shuanghua Baihe tablets significantly reduced the occurrence, severity, and latency of oral mucositis in patients with NPC during chemoradiotherapy. The overall incidence of gastric reactions associated with Shuanghua Baihe tablets was 3.33%. A randomized multicenter trial demonstrated that intravenous actovegin had positive effects on the treatment and prevention of chemoradiotherapy-induced oral mucositis in patients with NPC. Actovegin reduced the incidence of severe OM and decreased the occurrence of severe pain. Actovegin was injected intravenously five times per week during radiotherapy. Two patients withdrew from the study because of vomiting and fever. A multicenter, randomized controlled trial by Liang et al. (16) demonstrated that thalidomide (THD) reduced the incidence of OM but significantly increased the occurrence of constipation and dizziness, and intolerable dizziness caused 2.5% (2/80) of patients in the THD group to drop out of the study. A randomized controlled trial by Yang et al. (58) demonstrated that maxillofacial and oral massage (MOM) significantly attenuated the occurrence of severe radiotherapy-induced oral mucositis (SRTOM), and reduced oral pain, xerostomia, and dysphagia in patients with NPC; however, ≥ grade 3 adverse events were observed in 1.3% of patients during MOM. Weng et al.’s retrospective study (59) analyzed data for 463 patients with NPC with mucositis and found that antibiotics may be effective for the treatment of SRTOM during CRT, but may potentially adversely affect the prognosis (OS and DFS).

The severity of OM is generally associated with the grading score for oral pain. A prospective study by Hua et al. demonstrated that oxycodone (60) effectively reduced moderate-to-severe pain caused by oral mucositis in patients with NPC treated with CCRT. Grade 3 constipation (6.5% and 9.1%) and grade 3-4 vomiting (6.4% and 9.1%) were observed in both the moderate pain and severe pain groups. A prospective study by Guo et al. (61) showed that transdermal fentanyl (TDF) is effective in treating moderate-to-severe pain caused by oral mucositis in NPC patients undergoing chemoradiotherapy, with a 10.26% incidence of nausea and vomiting. Other studies have shown that some drugs, including THD (16), Shuanghua Baihe tablets (17), Actovegin (20), and maxillofacial and oral massage (58), could both prevent oral mucositis and reduce oral pain. In the present study, the most serious oral pain occurred during the severe mucositis phase, and the incidence of serious oral pain was decreased due to the reduced severity of oral mucositis by CBT, which was consistent with the results reported by Liang et al., Zheng et al., Wu et al., and Yang et al. (16, 17, 20, 58). The potential mechanism of CBT in reducing oral pain is that CBT reduces insomnia severity. Yang et al. (62) prospectively evaluated the effect of CBT on pain severity among cancer survivors (including head and neck cancer [HNC] patients) with comorbid pain and insomnia. The result showed that CBT led to pain reductions, possibly achieved by insomnia improvement. In a prospective trial conducted by Garland et al. (63), CBT produced clinically meaningful reductions in pain and insomnia severity in cancer survivors (including HNC patients). Our present study showed that CBT reduced insomnia and oral pain,which was consistent with Yang et al.’s and Garland et al.’s studies (58, 63).

### 4.1 Limitations

Our study had some limitations. First, it was conducted at a single center rather than at multiple centers, which may limit the generalizability of the findings. Second, due to the limited follow-up duration, late toxicities were not included in the analysis, which may have important implications for the sustained effectiveness of the intervention. Subsequent investigations incorporating extended follow-up periods and involving multiple medical centers are imperative to comprehensively assess the enduring effects of CBT as well as the potential occurrence of delayed adverse events.

## 5 Conclusions

Cognitive behavioral therapy reduced the incidence, latency, and severity of oral mucositis in patients with locoregional advanced nasopharyngeal carcinoma during concurrent chemoradiotherapy. Further follow-up and multicenter trials are needed to assess the long-term effects of CBT and late adverse events in NPC patients.

## Data availability statement

The original contributions presented in the study are included in the article/supplementary material. Further inquiries can be directed to the corresponding author.

## Ethics statement

The studies involving human participants were reviewed and approved by Ethics Committee of Hunan Cancer Hospital. The patients/participants provided their written informed consent to participate in this study. Informed consent was obtained from all participants included in the study during follow-up.

## Author contributions

FL designed the study, edited, and submitted the manuscript (FL is the corresponding author). FL, L-LH, SX, C-HJ, X-wW, WL, C-GF, XY, QZ, W-QW, Y-XL, and HW participated in chemotherapy, radiotherapy, treatment as usual, and cognitive behavioral therapy. LH and SX were involved in the design of the study, collection and analysis of the data, and drafting of the manuscript. All authors contributed to the article and approved the submitted version.
